# Watermelon goes viral: introducing a vector for virus-induced gene silencing in cucurbits

**DOI:** 10.1093/plphys/kiac438

**Published:** 2022-09-19

**Authors:** Yana Kazachkova

**Affiliations:** Department of Molecular Biology, Princeton University, Princeton, New Jersey 08544, USA

Plant male sterility is broadly defined as the inability to produce and release functional pollen. This phenomenon was first described more than 250 years ago and has been observed in over 600 plant species.

Male sterility is an important tool for breeders, facilitating the production of hybrid plants with higher yields. In self-pollinating species, it allows bypassing the need to manually emasculate flowers to prevent selfing (Chen and Liu, 2014). Hence, understanding the genetic mechanisms of male sterility is not only important from a basic biology perspective but is also beneficial for plant growers.

In watermelon (*Citrullus lanatus*), seven male-sterile mutants have been reported. However, only one of the mutant phenotypes has been linked to a specific gene mutation (Rhee et al., 2022). Overall, high-throughput functional characterization of genes in watermelon plants remains challenging due to the requirement for a prolonged growth period and large area for plant cultivation.

Usually, characterization of gene function requires the generation of stable transgenic plants, either by particle bombardment or via *Agrobacterium*-mediated transformation. Particle bombardment employs DNA-coated metal microparticles that are shot at target cells or tissues using pressurized gas. Inside the cell, DNA elutes from the particles, a fraction of which integrates into the plant genome (Kikkert et al., 2004). *Agrobacterium*-mediated transformation harnesses the ability of soil-borne *Agrobacterium tumefaciens* to integrate a region of its tumor-inducing plasmid into the plant genome (Sharma et al., 2005). Independent of the transformation method, the foreign DNA permanently integrates into the plant genome and is inherited by the next generations.

Stable transformation takes a substantial amount of time and remains challenging for many plant species (Sharma et al., 2005). Although several reports show successful production of stable knock-out mutants in cucurbits, *Cucurbitaceae* plants show very low transformation efficiency (Tian et al., 2017; Wang et al., 2021). Hence, a rapid method for screening gene loss-of-function is valuable to complement plant stable transformation.

Virus-induced gene silencing (VIGS) is an RNA interference-based technology used to transiently knock-down target gene expression. VIGS is based on post-transcriptional gene silencing, employing a plant defense system against RNA viruses (Dommes et al., 2019). A short 200–300 base pair fragment of the target gene is cloned into a plasmid containing a viral sequence. Double-stranded RNA, produced during the virus replication cycle in the infected plant cell, is degraded by DICER proteins to form small interfering RNAs. These are incorporated into an RNA-induced silencing complex that targets mRNA molecules with the complementary sequence, leading to their degradation (Courdavault et al., 2020). VIGS has been successfully used in tomato (*Solanum lycopersicum*), strawberry (*Fragaria ananassa*), *Nicotiana benthamiana*, rice (*Oryza sativa*), corn (*Zea mays*), and barley (*Hordeum vulgare*) to silence genes in leaves, flowers, and fruits (Dommes et al., 2019).

In this issue of *Plant Physiology*, Sun-Ju Rhee and colleagues describe the VIGS vector pCF93 based on the *Cucumber fruit mottle mosaic virus* (CFMMV) where the production of viral RNAs is driven by the 35S promoter (Rhee et al., 2022). The authors first selected the most efficient VIGS vector for functional gene characterization by testing 4 out of 14 previously engineered CFMMV vectors (Rhee et al., 2016). The authors cloned a fragment of the *PHYTOENE DESATURASE* (*PDS*) gene as a visual marker of silencing into each vector and observed the photobleaching phenotype in *N. benthamiana* leaves 12–15 days after inoculation. Infection with pCF93 resulted in the largest photobleached leaf area compared with the other tested vectors. Hence, the authors selected this vector for further experiments.

The authors then infected cotyledons of cucumber (*Cucumis sativus*), melon (*Cucumis melo*), and watermelon using the pCF93 vector containing *PDS* fragments specific for each species. Interestingly, pCF93-*PDS* not only successfully silenced *PDS* in newly developed leaves but also in plant reproductive organs.

Rhee and colleagues tested *PDS* silencing in three small-fruited watermelon cultivars that can be grown in pots in the greenhouse. When harvested, all watermelon fruits had lighter peels and white flesh due to chlorophyll degradation and low lycopene and β-carotene levels. The DAH cultivar showed the most prominent silencing phenotype and was selected for future analysis.

Next, they used this optimized system to identify genes involved in male sterility. The authors used previously published transcriptome data to pinpoint 38 differentially expressed genes between male-fertile and male-sterile accessions of watermelon. They amplified a fragment of each gene, cloned the fragments individually into separate pCF93 vectors, and inoculated 550 DAH watermelon seedlings to study the function of each gene. Eight out of 38 genes induced male sterility of varying severity, ranging from complete to moderate male-sterile phenotypes. In complete male-sterile flowers, the stamens were dramatically smaller and were green and immature with no pollen (Figure 1). Partially male-sterile flowers had one or two abnormal stamens, but the remaining stamens were able to produce pollen. Histological analysis showed that the mature flowers had developmental defects and abnormal pollen sacks similar to the previously characterized male-sterile cultivar DAH3615-MS. Hence, VIGS in watermelon allowed replication of male-sterility phenotypes to circumvent the need to produce stable transgenic lines. Moreover, the use of a small watermelon cultivar allowed experiments to be carried out in pots in the greenhouse.

**Figure 1:**
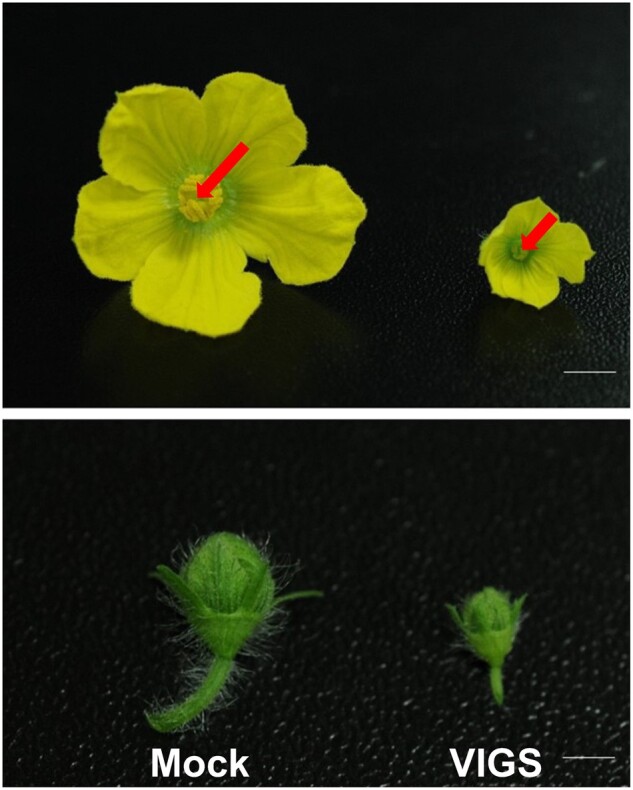
Top and side views of mock and male-sterile flowers on watermelon plants silenced for candidate genes via VIGS. Plants were inoculated with the pCF93 viral vector harboring a ca. 300 bp fragment of one of the candidate genes. For mock treatment, plants were infected with the pCF-*PDS-*int plasmid. Some of the silenced flowers exhibited a complete male sterility phenotype, while others exhibited varying degrees of male sterility. An example of a complete male-sterility phenotype from the plant with silenced *expansin A-9* is shown on the right while the representative mock flower is depicted on the left. Red arrow indicates the visible yellow stamen in mock-treated flower and the absence of the stamen in the male-sterile flower. Figure 4 from the original manuscript was used to create this figure. Scale bars, 0.5 cm.

In their work, Rhee and colleagues showed that the pCF93 vector and the small watermelon cultivar-based VIGS system will be extremely valuable for high-throughput gene function analysis in watermelon and other cucurbits.

*Conflict of interest statement*. None declared.

## References

[kiac438-B1] Chen L , Liu YG (2014) Male sterility and fertility restoration in plants. Annu Rev Plant Biol 65: 579–606 24313845 10.1146/annurev-arplant-050213-040119

[kiac438-B2] Courdavault V , Besseau S, Oudin A, Papon N, O’Connor SE (2020) Virus-induced gene silencing: Hush genes to make them talk. Trends Plant Sci 25: 714–715 32526175 10.1016/j.tplants.2020.02.013

[kiac438-B3] Dommes AB , Gross T, Herbert DB, Kivivirta KI, Becker A (2019) Virus-induced gene silencing: Empowering genetics in non-model organisms. J Exp Bot 70: 757–770 30452695 10.1093/jxb/ery411

[kiac438-B4] Kikkert JR , Vidal JR, Reisch BI (2004) Stable transformation of plant cells by particle bombardment/biolistics. *In* Peña L, ed, Transgenic Plants: Methods and Protocols. Humana Press, Totowa, NJ, pp 61–78

[kiac438-B5] Rhee SJ , Jang YJ, Lee GP (2016) Identification of the subgenomic promoter of the coat protein gene of cucumber fruit mottle mosaic virus and development of a heterologous expression vector. Archiv Virol 161: 1527–1538 10.1007/s00705-016-2808-9 26976138

[kiac438-B6] Rhee S-J , Jeong Jang Y, Park J-Y, Ryu J, Pyo Lee G (2022) Virus-induced gene silencing for *in planta* validation of gene function in cucurbits. Plant Physiol **190**: 2366–2379 10.1093/plphys/kiac363 PMC9706489 35944218

[kiac438-B7] Sharma KK , Bhatnagar-Mathur P, Thorpe TA (2005) Genetic transformation technology: Status and problems. In Vitro Cell Dev Biol Plant 41: 102–112

[kiac438-B8] Tian S , Jiang L, Gao Q, Zhang J, Zong M, Zhang H, Ren Y, Guo S, Gong G, Liu F, et al (2017) Efficient CRISPR/Cas9-based gene knockout in watermelon. Plant Cell Rep 36: 399–406 27995308 10.1007/s00299-016-2089-5

[kiac438-B9] Wang Y , Wang J, Guo S, Tian S, Zhang J, Ren Y, Li M, Gong G, Zhang H, Xu Y (2021) CRISPR/Cas9-mediated mutagenesis of ClBG1 decreased seed size and promoted seed germination in watermelon. Horticult Res 8: 70 10.1038/s41438-021-00506-1 PMC8012358 33790265

